# Provider-ordered viral testing and antibiotic administration practices among children with acute respiratory infections across healthcare settings in Nashville, Tennessee

**DOI:** 10.1017/ash.2024.24

**Published:** 2024-03-06

**Authors:** Danielle A. Rankin, Sophie E. Katz, Justin Z. Amarin, Haya Hayek, Laura S. Stewart, James C. Slaughter, Stephen Deppen, Ahmad Yanis, Yesenia Herazo Romero, James D. Chappell, Nikhil K. Khankari, Natasha B. Halasa

**Affiliations:** 1 Department of Pediatrics, Vanderbilt University Medical Center, Nashville, TN, USA; 2 Vanderbilt Epidemiology PhD Program, Vanderbilt University School of Medicine, Nashville, TN, USA; 3 Department of Biostatistics, Vanderbilt University Medical Center, Nashville, TN, USA; 4 Department of Thoracic Surgery and Division of Epidemiology, Vanderbilt University Medical Center, Nashville, TN, USA; 5 Division of Genetic Medicine, Vanderbilt University Medical Center, Nashville, TN, USA

## Abstract

**Objective:**

Evaluate the association between provider-ordered viral testing and antibiotic treatment practices among children discharged from an ED or hospitalized with an acute respiratory infection (ARI).

**Design:**

Active, prospective ARI surveillance study from November 2017 to February 2020.

**Setting:**

Pediatric hospital and emergency department in Nashville, Tennessee.

**Participants:**

Children 30 days to 17 years old seeking medical care for fever and/or respiratory symptoms.

**Methods:**

Antibiotics prescribed during the child’s ED visit or administered during hospitalization were categorized into (1) None administered; (2) Narrow-spectrum; and (3) Broad-spectrum. Setting-specific models were built using unconditional polytomous logistic regression with robust sandwich estimators to estimate the adjusted odds ratios and 95% confidence intervals between provider-ordered viral testing (ie, tested versus not tested) and viral test result (ie, positive test versus not tested and negative test versus not tested) and three-level antibiotic administration.

**Results:**

4,107 children were enrolled and tested, of which 2,616 (64%) were seen in the ED and 1,491 (36%) were hospitalized. In the ED, children who received a provider-ordered viral test had 25% decreased odds (aOR: 0.75; 95% CI: 0.54, 0.98) of receiving a narrow-spectrum antibiotic during their visit than those without testing. In the inpatient setting, children with a negative provider-ordered viral test had 57% increased odds (aOR: 1.57; 95% CI: 1.01, 2.44) of being administered a broad-spectrum antibiotic compared to children without testing.

**Conclusions:**

In our study, the impact of provider-ordered viral testing on antibiotic practices differed by setting. Additional studies evaluating the influence of viral testing on antibiotic stewardship and antibiotic prescribing practices are needed.

## Introduction

In the United States, 12 to 32 million acute respiratory infection (ARI) episodes are reported in infants (<1 year old) and nearly 200 million in children (≥1 year old) per year^1^ accounting for 41% of emergency department (ED) visits and 40% of hospitalizations.^2^ Since the introduction of pneumococcal and *Hemophilus influenzae* vaccines, viruses have become the primary cause of ARIs,^3^ but bacterial infections still occur, making clinical diagnosis and management challenging. Children with ARIs often present with fever or shortness of breath, often leading to empiric antibiotic therapy—typically broad-spectrum—and diagnostic testing.^4^ Mounting evidence has shown broad-spectrum antibiotics are used in situations where narrow-spectrum antibiotics or no antibiotics are warranted (inappropriate antibiotic use), with prescribing rates highest across Southern ambulatory care settings (ie, EDs and outpatient clinics).^5–9^

Advancements in rapid diagnostic viral testing have made testing options more accurate, faster, less expensive, and more readily accessible.^4,10,11^ However, the impact of viral diagnostic testing on antibiotic administration for children with ARI remains unclear due to conflicting results, with some studies reporting that viral diagnostics are associated with lower antibiotic use, while others report no impact.^12,13^ Likewise, studies examining viral testing and antibiotic prescribing and administration practices in both EDs and inpatient settings are limited. Therefore, in this prospective study, we aimed to evaluate the association of (1) provider-ordered viral testing and antibiotic treatment, and (2) provider-ordered viral test result (ie, positive, negative) and antibiotic treatment among children presenting to an ED or hospitalized with an ARI at major tertiary care children’s hospital in Nashville, Tennessee. We hypothesized that a provider-ordered test, regardless of the result, would be associated with reduced antibiotic treatment among children in both the ED and inpatient settings. Provider-ordered viral testing and antibiotic treatment were further evaluated to assess for therapeutic appropriateness by CDC’s Tiered Discharge Diagnosis.^6^

## Methods

### Study design

We conducted a secondary analysis using data collected from November 2, 2017, to February 28, 2020, from the New Vaccine Network (NVSN). NVSN is an ongoing multi-center, active, prospective ARI surveillance study in children (<18 years) funded by the Centers for Disease Control and Prevention (CDC).^14,15^ This study only includes data collected at the Nashville, Tennessee site. Year-round recruitment occurred 4 days per week in the ED and 7 days per week in the inpatient setting at the Monroe Carell Jr. Children’s Hospital at Vanderbilt University Medical Center (VUMC). Institutional Review Boards at VUMC and the CDC reviewed and approved this study.^16^

### Study population

Children were eligible if they presented to an ED or were hospitalized within 48 hours of presentation and had at least one of the following symptoms in the 14 days prior to enrollment: fever, cough, earache, nasal congestion, runny nose, sore throat, post-tussive vomiting, wheezing, shortness of breath, rapid or shallow breathing, myalgia, apnea, apparent life-threatening event, or brief resolved unexplained event (eg, unresponsive, etc.).^14,15^ In addition, eligible children had to live within the catchment/surveillance area (eg, Davidson, Williamson, Cheatham, Dickson, Rutherford, Montgomery, Roberson, and Sumner Counties). Children were excluded if they met any of the following criteria: chemotherapy-associated fever and neutropenia (absolute neutrophil count <500 × 10^3^/µL), newborns never discharged since birth, transferred to VUMC from another hospital >48 hours after initial admission, recently hospitalized (≤5 days), or previously enrolled in the study within the past 14 days.^14,15^

We also excluded children younger than 30 days to minimize the inclusion of serious bacterial infections due to differences in clinical management practices, and children with multiple ARI enrollments <90 days apart. We considered children with multiple ARI enrollments (≥90 days from the prior visit) as susceptible to a new viral or bacterial ARI, thus increasing the potential for additional provider-ordered viral testing and antibiotic use.

### Illness history, clinical characteristics, and provider-ordered testing

After obtaining informed consent, parents/guardians of enrolled children were interviewed by trained research personnel to collect demographic (age, sex, race, and ethnicity) information, parent-reported clinical symptoms (eg, fever, myalgia, lethargy, etc.), and past medical history using a standardized form. Provider-ordered viral testing (ie, influenza antigen, respiratory syncytial virus (RSV) antigen, and molecular respiratory testing), vital signs at time of presentation, physical exam findings, other clinical lab testing (ie, rapid *Streptococcus* antigen testing and bacterial cultures), the first five discharge diagnoses (*International Classification of Diseases, Tenth Revision* [ICD-10]), and active and completed antibiotics were abstracted from each child’s medical record. Final clinical disposition was determined by the highest level of care provided to the child during the visit (ie, children enrolled in the ED and later hospitalized were considered inpatient). At VUMC, molecular respiratory testing is performed using BioFire® FilmArray Respiratory Pathogen Panel 2.0 and consists of the following targets: adenovirus, non-pandemic coronavirus strains (229E, HKU1, NL63, OC43), human metapneumovirus, influenza, parainfluenza (types 1–4), rhinovirus/enterovirus, RSV, and respiratory bacterial pathogens (ie, *Bordetella pertussis, Chlamydophilia pneumoniae, Legionella pneumophilia,* and *Mycoplasma pneumoniae*).^17,18^ The exposure variable was defined as receipt of at least one provider-order viral test, including rapid influenza antigen, rapid RSV antigen, and/or molecular, dichotomized as tested versus not tested (referent).

Interview, medical record, and laboratory results were maintained in a secure REDCap™ (Research Electronic Data Capture, Vanderbilt University, Nashville, TN, USA) database.^19^

### Antibiotic administration and tiered discharge diagnoses

We defined our outcome variable as antibiotic prescribed during the child’s ED visit or administered during hospitalization. Antibiotics were categorized in three main groups: (1) None administered; (2) Narrow-spectrum (eg, penicillins, tetracyclines, first-generation cephalosporins, and sulfonamides); and (3) Broad-spectrum [eg, macrolides (azithromycin), amoxicillin-clavulanate, advanced-generation cephalosporins, quinolones, and clindamycin].^20,21^ In the instance both narrow- and broad-spectrum antibiotics were administered/prescribed during the child’s healthcare visit/stay, broad-spectrum was assigned (eTable 1 in Supplement).

We classified ICD-10 discharge diagnoses based on the indication for an antibiotic using CDC’s tiered diagnosis system (Tier 1—antibiotics almost always indicated; Tier 2—antibiotics may be indicated; Tier 3—antibotics are not indicated or indications are unclear).^6^ If a child’s visit had ICD-10 codes in multiple tiers, priority was given to Tier 1 diagnoses, followed by Tier 2 and Tier 3 diagnoses.^6^ Tiered diagnoses were further compared to antibiotic treatment and provider-ordered viral testing in healthcare setting.

### Statistical analysis

We summarized children’s sociodemographic and clinical characteristics by setting using frequency (percent) for categorical variables and mean (standard deviation) for continuous variables. We performed multiple imputations for 213 children with ≥1 missing covariate information, using predictive mean matching with 10 imputation samples.^22^

For each healthcare setting, confounders were assessed through directed acyclic graphs (eFigure 1 in Supplement**)** generated using prior knowledge. Setting-specific models were built using unconditional polytomous logistic regression with robust sandwich estimators (to account for repeat enrollments) to estimate adjusted odds ratios (aORs) and 95% confidence intervals between provider-ordered viral testing (ie, tested versus not tested) and provider-ordered viral test result (ie, positive test versus not tested and negative test versus not tested) and three-level antibiotic administration (ie, no antibiotics prescribed/administered [referent], narrow-spectrum, or broad-spectrum). Restricted cubic splines were generated for continuous covariates (eTable 2 in Supplement). Models were adjusted for parent-reported pulmonary symptoms (cough, nasal congestion, rhinorrhea, wheezing, and shortness of breath; yes or no), provider documented wheezing (yes or no), retractions (yes or no), time of year (respiratory season [October–April] or non-respiratory season), any underlying medical condition (yes or no), insurance (none/self-pay, private, or public), temperature [none, moderate (≥100.4°F to <102.0°F), or severe (≥102.0°F)], age (years), heart rate (beats/minute), respiratory rate (breaths/minute), oxygen saturation (capillary), illness duration (days), and multiplicative interactions between age and heart rate and age and respiratory rate. We performed an exploratory analysis evaluating the impact of viral/bacterial pathogen detected and the receipt of antibiotics. Statistical tests were based on two-tailed probability with a significance level (α) set at 5%. All analyses were performed in R (version 3.6.1).

## Results

From November 2017 to February 2020, we enrolled 4,107 children, of which 2,616 (64%) were from the ED and 1,491 (36%) were hospitalized (eFigure 2 in Supplement). Of the 4,107 enrollments, 455 (11%) children represented multiple ARI enrollments ≥90 days apart, which were not confined to one healthcare setting. Overall, 55% (2,249/4,107) of children received at least one provider-ordered viral test, with 49% (1,294/2,616) and 64% (955/1,491) of children tested in the ED and inpatient setting, respectively (Table 1).

**Table 1.** Sociodemographic and clinical characteristics of children (>30 d–17 yr) enrolled in an emergency department and hospitalized with acute respiratory illness, by provider-ordered viral testing, Nashville, Tennessee, November 2017–February 2020

Characteristic(s)	Emergency Department		Inpatient	
	Viral Test Ordered N = 1,294	No Viral Test Ordered N = 1,322	Viral Test Ordered N = 955	No Viral Test Ordered N = 536
Age, years—mean (SD)	4.8 (4.5)	4.4 (4.3)	3.2 (4.1)	3.8 (4.2)
Age, years—median (IQR)	3.3 (1.2, 7.3)	2.8 (1.0, 6.5)	1.4 (0.4, 4.1)	2.0 (0.6, 6.0)
0 to <2 yr—no. (%)	483 (37.3)	545 (41.2)	565 (59.2)	267 (49.8)
2 to <5 yr—no. (%)	321 (24.8)	341 (25.8)	181 (19.0)	112 (20.9)
≥5 to <18 yr—no. (%)	490 (37.9)	436 (33.0)	209 (21.9)	157 (29.3)
Sex, male—no. (%)	690 (53.3)	715 (54.1)	552 (57.8)	321 (59.9)
*Race—no. (%)*				
White, Non-Hispanic	172 (13.3)	181 (13.7)	436 (45.7)	240 (44.9)
Black, Non-Hispanic	517 (40.0)	598 (45.2)	221 (23.1)	134 (25.0)
Other Non-Hispanic	84 (6.5)	98 (7.4)	90 (9.4)	45 (8.4)
Hispanic	509 (39.3)	431 (32.6)	197 (20.6)	113 (21.1)
Unknown	12 (0.9)	14 (1.1)	11 (1.2)	4 (0.7)
*Insurance type—no. (%)*				
Public	1,049 (81.1)	1,044 (79.0)	630 (66.0)	338 (63.1)
Private	104 (8.0)	115 (8.7)	149 (27.8)	246 (25.8)
Both	6 (0.5)	7 (0.5)	12 (2.2)	15 (1.6)
None/Self Pay	121 (9.1)	141 (10.7)	31 (5.8)	49 (5.1)
Unknown	14 (1.1)	15 (1.1)	6 (1.1)	15 (1.6)
*Underlying medical conditions—no. (%)*				
≥1 Underlying medical condition	415 (32.1)	454 (34.3)	492 (51.5)	273 (50.9)
Respiratory	225 (17.4)	246 (18.6)	242 (25.3)	164 (30.6)
Asthma/RAD, only	206 (15.9)	227 (17.2)	154 (16.1)	138 (25.7)
Cardiovascular	28 (2.2)	24 (1.8)	104 (10.9)	23 (4.3)
Neurological/Neuromuscular	65 (5.0)	55 (4.2)	168 (17.6)	52 (9.7)
Oncologic/Immunosuppressive	141 (10.9)	177 (13.4)	158 (16.5)	109 (20.3)
Renal	2 (0.2)	2 (0.2)	11 (1.2)	4 (0.7)
Gastrointestinal/Hepatic	39 (3.0)	25 (1.9)	127 (13.3)	29 (5.4)
Endocrine	5 (0.4)	4 (0.3)	28 (2.9)	10 (1.9)
Other	20 (1.5)	56 (4.2)	61 (6.4)	27 (5.0)
Prematurity, <37 wk^a^	63 (4.9)	46 (3.5)	107 (11.2)	37 (6.9)
*Time of Illness* ^b^ *—no. (%)*				
Respiratory season	1,150 (88.9)	852 (64.4)	766 (80.2)	382 (71.3)
Antibiotic consumption ≤14 d prior to visit/hospitalization—no. (%)	66 (5.1)	92 (7.0)	171 (17.9)	86 (16.0)
Illness duration, d (mean [SD])	2.6 (2.1)	3.0^c^ (2.4)	3.4^d^ (2.6)	3.3 (2.5)
*Vital Signs at presentation—mean (SD)*				
Maximum temperature, °F (mean [SD])	101.9^e^ (2.0)	100.6^f^ (2.1)	101.5^g^ (2.2)	100.8^h^ (2.1)
Heart rate, bpm	139^i^ (28.6)	132.3^j^ (28.0)	152.1^k^ (27.4)	147 (26.7)
Respiratory rate, bpm	28^l^ (8.7)	29^m^ (9.8)	38.4^k^ (13.5)	38.7 (14.2)
Oxygen Saturation, capillary	98.4^n^ (1.9)	98.4^o^ (1.9)	95.3 (5.9)	95.7 (3.8)
*Physical assessment—no. (%)*				
Wheezing	89 (6.9)	189 (14.3)	289 (30.3)	250 (46.6)
Retractions	67 (5.2)	165 (12.5)	460 (48.2)	307 (57.3)
Rapid *Streptococcus* antigen, positive—no. (%)	77 (6.0)	69 (5.2)	10 (1.0)	5 (0.9)
*Clinical Laboratory Cultures—no. (%)*				
Bacterial culture, positive	39 (3.0)	29 (2.2)	67 (7.0)	14 (2.6)
Blood	0	0	3 (0.3)	0
Cerebral spinal fluid	0	1 (0.1)	9 (0.9)	2 (0.4)
Respiratory	23 (1.8)	11 (0.8)	19 (2.0)	6 (1.1)
Urine	14 (1.1)	13 (1.0)	33 (3.5)	4 (0.7)
Other	2 (0.2)	4 (0.3)	3 (0.3)	2 (0.4)

a History of prematurity was reported for children aged <2 yr.

b Respiratory season, October–April.

c n = 1,321.

d n = 953.

e n = 1,219

f n = 1,261.

g n = 923

h n = 522

i n = 1,290.

j n = 1,313.

k n = 954.

l n = 1,288.

m n = 1,314.

n n = 1,289.

o n = 1,316.

### Patient characteristics, by setting

No sociodemographic differences between receiving and not receiving a provider-ordered viral test were observed among children in either setting (Table 1). Overall, children were predominately younger than 5 years and had public health insurance (ie, Medicaid). In the ED, children were predominantly Black, non-Hispanic, and approximately one-third reported at least one underlying medical condition. Children hospitalized were primarily White, non-Hispanic, and about one-half had a known underlying medical condition, with respiratory conditions being the most common. In both settings, provider-ordered tests during respiratory season (October-April) were more frequent than in non-respiratory season. In contrast, for children seen in the ED and hospitalized with an ARI, a provider-ordered test was less commonly ordered if wheezing and retractions were found on physical examination (Table 1).

**Figure 1.**
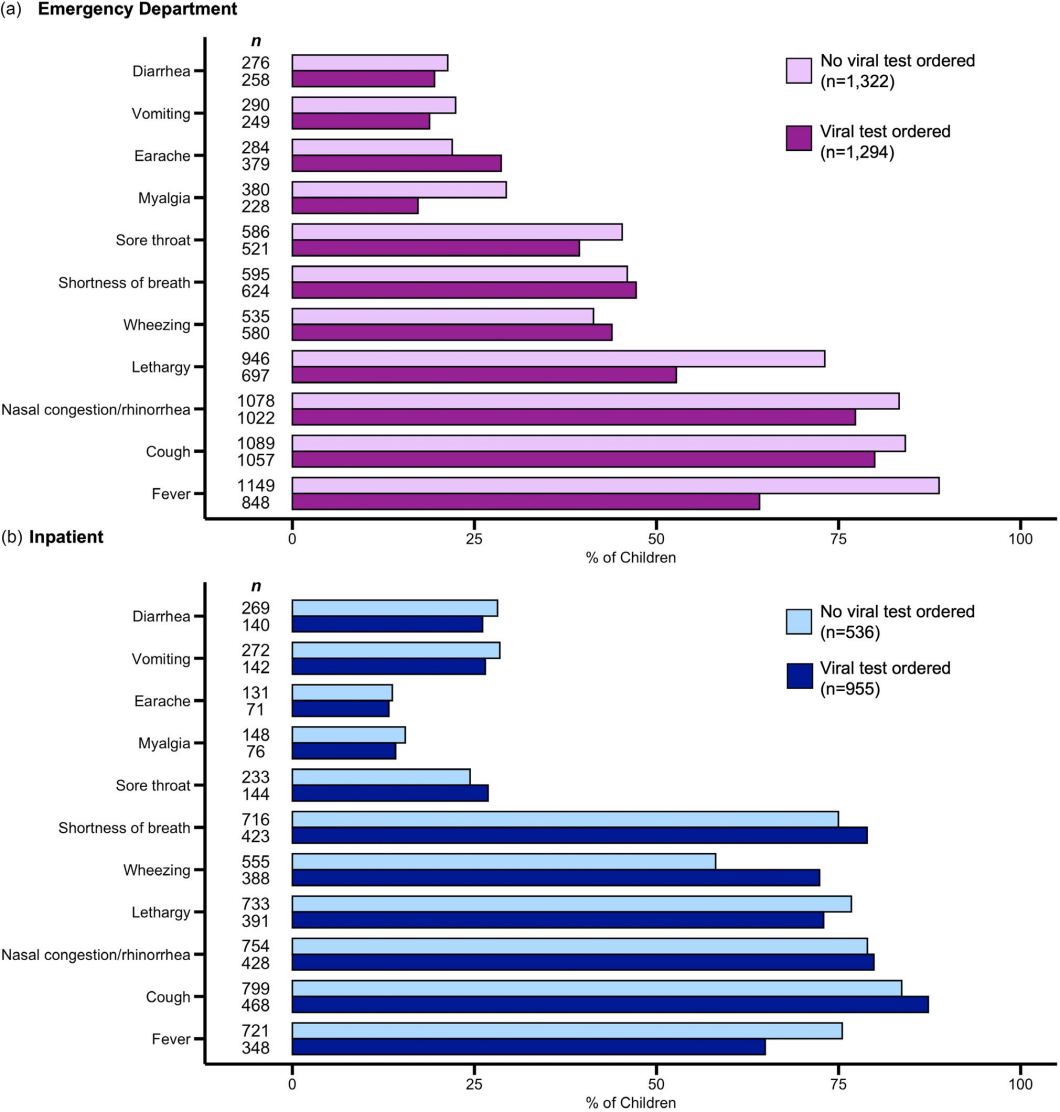
Symptom presentation of children (>30 days–17 years) enrolled in an emergency department and hospitalized with acute respiratory illness, by clinical provider-ordered testing, Nashville, Tennessee, November 2017–February 2020.

Children seen in the ED most often presented with fever, cough, and nasal congestion/rhinorrhea. Those who presented with myalgia, lethargy, or fever were less likely to have provider-ordered viral testing (Figure 1A). Among hospitalized children, cough, nasal/congestion, lethargy, and shortness of breath were the most common symptoms (Figure 1B); more patients with fever had a provider-ordered viral test (75% vs 64%, respectively). Of note, parent-reported symptoms in Figure 1 are not mutually exclusive.

### Provider-ordered testing, by setting

Children with and without provider-ordered viral tests were more often given a rapid *Streptococcus* antigen test in the ED (22%; 573/2,616) than in the inpatient setting (5%; 68/1,491). Among children seen in the ED, no differences in clinical culture orders were observed between those with and without provider-ordered viral testing. Compared to children hospitalized without a provider-ordered viral test, those with viral testing were more likely to also have bacterial cultures performed, of which 7% detected a bacterial pathogen (Table 1). Bacterial pathogens were most frequently identified from urine and respiratory (eg, sputum, throat) sources in both the ED and inpatient settings.

In the ED, 87% (1,126/1,294) of viral tests were a rapid influenza antigen, whereas 73% (698/955) of tests in children hospitalized were a molecular respiratory panel (Table 2). The two most common viral pathogens detected from molecular testing were rhinovirus/enterovirus and parainfluenza in the ED and rhinovirus/enterovirus and RSV in the inpatient setting.

**Table 2.** Provider-ordered viral testing results among children (>30 d–17 yr) with acute respiratory illness, by setting, Nashville, Tennessee, November 2017–February 2020

Provider-ordered viral testing^a^	Emergency department (n = 1,294) no. (%)	Inpatient (n = 955) no. (%)
Any Provider-Ordered test, positive	854 (66.0)	694 (72.7)
*Rapid Antigen Test(s)*		
Rapid influenza antigen performed	1,126 (87.0)	339 (35.5)
Rapid influenza antigen, positive	701/1,126 (62.3)	213/339 (62.8)
Rapid RSV antigen performed	28 (2.2)	117 (12.3)
Rapid RSV antigen, positive	16/28 (57.1)	64/117 (12.3)
*Molecular Test(s) performed*	180 (13.9)	698 (73.1)
Adenovirus	20/180 (11.1)	58/698 (8.3)
Influenza	17/180 (9.4)	51/698 (7.3)
RSV	23/180 (12.8)	150/698 (21.5)
Rhinovirus/enterovirus	63/180 (35.0)	224/698 (32.1)
Parainfluenza (Types 1–4)	29/180 (16.1)	45/698 (6.4)
Human metapneumovirus	8/180 (4.4)	45/698 (6.4)
Coronaviruses (endemic)	16/180 (8.9)	35/698 (5.0)
*Mycoplasma pneumoniae*	2/180 (1.1)	21/698 (3.0)
*Bordetella parapertussis*	0	0
*Legionella pneumophilia*	0	0
*Chlamydophilia pneumoniae*	0	0

a Multiple provider-ordered tests may have been administered to a single child during their visit/hospitalization.

### Provider-ordered viral testing and antibiotic administration

Antibiotics were prescribed in 16% (201/1,294; 74% narrow-spectrum; 26% broad-spectrum) and 18% (231/1,322; 72% narrow-spectrum; 28% broad-spectrum) of children with and without a provider-ordered test in the ED, respectively. Among children hospitalized, antibiotics were administered in 25% (239/955; 25% narrow-spectrum; 75% broad-spectrum) of children hospitalized with viral testing and 18% (95/536; 35% narrow-spectrum; 65% broad-spectrum) without viral testing.

In the ED, receipt of provider-ordered viral testing, regardless of the result (eg, positive or negative), resulted in a decreased odds of narrow-spectrum antibiotic prescriptions. Specifically, children who received a provider-ordered viral test had 25% lower odds (aOR: 0.75; 95% CI: 0.54, 0.98) of being prescribed a narrow-spectrum antibiotic than no antibiotic during their visit than those who did not receive a viral test (Table 3). Conversely prescription of broad-spectrum antibiotics was dependent on the result of the provider-ordered viral test, where we observed a positive result led to a decrease in broad-spectrum therapy, but a negative result showed an increase in broad-spectrum therapy. However, results for these estimates were imprecise to draw strong inferences. There was no difference in antibiotic administration practices based on the pathogen detected from the provider-ordered test (eTable 3 in Supplement).

**Table 3.** Polytomous logistic regression of the association of provider-ordered viral testing and antibiotic administration among children enrolled in an emergency department and hospitalized with acute respiratory illness, November 2017–February 2020

					Antibiotics					
Setting	Provider-Ordered Viral Test	No Antibiotic(s)	aOR (95% CI)	*P* value	Narrow-Spectrum	aOR (95% CI)	*P* value	Broad-Spectrum	aOR (95% CI)	*P* value
ED (n = 2,616)	Viral Test/No Viral Test	1093/1091	Ref.	Ref.	148/167	0.75 (0.54, 0.98)	0.035	53/64	0.89 (0.58, 1.36)	0.577
	Positive Viral Test/No Viral Test	723/1091	Ref.	Ref.	101/167	0.75 (0.56, 1.01)	0.062	30/64	0.72 (0.44, 1.19)	0.205
	Negative Viral Test/No Viral Test	370/1091	Ref.	Ref.	47/167	0.74 (0.51, 1.07)	0.111	23/64	1.11 (0.65, 1.89)	0.696
Inpatient (n = 1,491)	Viral Test/No Viral Test	716/441	Ref.	Ref.	60/33	1.00 (0.62, 1.62)	0.989	179/62	1.27 (0.90, 1.80)	0.180
	Positive Viral Test/No Viral Test	538/441	Ref.	Ref.	41/33	0.90 (0.53, 1.51)	0.683	115/62	1.16 (0.79, 1.68)	0.448
	Negative Viral Test/No Viral Test	178/441	Ref.	Ref.	19/33	1.34 (0.71, 2.53)	0.369	64/62	1.57 (1.01, 2.44)	0.046

Note. All models were adjusted for pulmonary symptoms (cough, nasal congestion, rhinorrhea, self-reported wheezing, and shortness of breath; yes or no); age (restricted cubic spline, 4-knots), maximum temperature (<100.4°F, ≥100.4°F -<102.0°F, ≥102.0°F), illness duration (restricted cubic spline, 3-knots), respiratory rate (restricted cubic spline, 4-knots), heart rate (restricted cubic spline, 4-knots), oxygen saturation (restricted cubic spline, 3-knots), wheezing (yes or no), retractions (yes or no), insurance (none/self-pay, private, or public), underlying medical condition (yes or no); time of year [respiratory season (October–April) or non-respiratory season]; age*heart rate (restricted cubic spline, 4-knots each), age*respiratory rate (restricted cubic spline, 4-knots each).

In the inpatient setting, receipt of provider-ordered testing had no effect on narrow-spectrum antibiotics; but a positive test showed a slightly lower odds of narrow-spectrum therapy, where a negative result indicated higher odds of narrow-spectrum therapy. Strong inferences from these estimates were unable to be drawn due to imprecision. Overall, regardless of the viral test results, receipt of provider-ordered viral test resulted in increased odds of broad-spectrum antibiotic administration. Children with a negative provider-ordered viral test had 57% increased odds (aOR: 1.57; 95% CI: 1.01, 2.44) of being administered a broad-spectrum antibiotic compared to children with no viral testing (Table 3). There was no difference in antibiotic administration practices based on the pathogen detected from the provider-ordered test (eTable 3 in Supplement).

**Figure 2.**
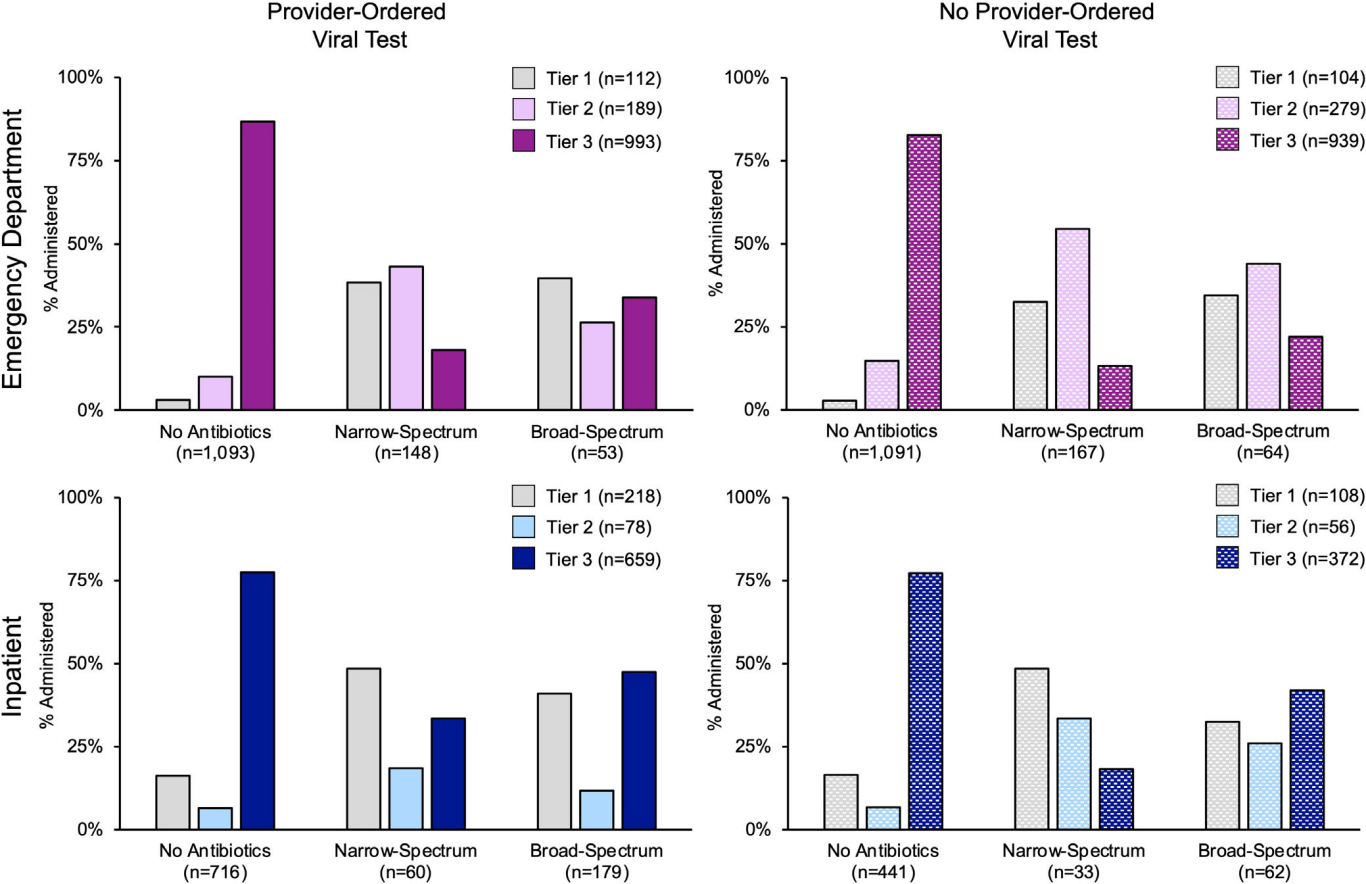
Antibiotic Administration Practices Among Children (>30 days–17 years) Enrolled in an Emergency Department and Hospitalized with Acute Respiratory Illness, by Tiered Discharge Diagnosis Classification and Provider-Ordered Viral Testing, Nashville, Tennessee, November 2017–February 2020. Footnote: Tier 1-antibiotics almost always indicated; Tier 2-antibiotics may be indicated; Tier 3-antibiotics are not indicated or indications are unclear.^6^

Administration of antibiotics by tiered discharge diagnoses and setting are shown in Figure 2. In the ED, 74% (1,932/2,616) of children had a tier 3 discharge diagnosis, with unspecified fever (467/1,932; 24%) and unspecified acute upper respiratory infection (409/1,932; 21%) as the two most common diagnoses. In tiers 1 and 2, *Streptococcal* pharyngitis (125/283; 58%) and acute otitis media (214/463; 46%) were the most frequent diagnoses, respectively. Among children in the ED with a tier 3 diagnosis, 96% were not prescribed an antibiotic, regardless of provider-ordered viral testing status. Narrow-spectrum (73%; 315/432) antibiotics were most frequently prescribed in the ED, with 35% of prescriptions in children with a tier 1 diagnosis, 50% in tier 2, and 16% in tier 3 (Figure 2).

Among hospitalized children, 72% (1,077/1,491) were assigned a tier 3 diagnosis, followed by tier 2 and then tier 1 (Figure 2). The most common discharge diagnoses were pneumonia (73/275; 27%), acute otitis media (81/139; 58%), and acute bronchiolitis due to RSV (195/1,077; 18%) for tiers 1, 2, and 3, respectively. Overall, 87% of children hospitalized and assigned a tier 3 discharge diagnosis were not administered an antibiotic. In the inpatient setting, broad-spectrum (72%; 241/334) were the most common antibiotics administered, with 39% of broad-spectrum antibiotics administered to patients with discharge diagnoses in tier 1, 15% in tier 2, and 46% in tier 3. Of note, broad-spectrum antibiotic administration was higher among children with a tier 1 or tier 3 diagnosis and a provider-ordered viral test than children with the same tiered diagnosis and no provider-ordered viral test (Figure 2).

## Discussion

In this active ARI surveillance study of 4,107 children in Nashville, Tennessee, we evaluated the association of provider-ordered viral testing and test results with antibiotic prescribing and administration in the ED and inpatient settings. In the ED, influenza rapid antigen tests were administered in 87% of all ARI visits. The most common antibiotics prescribed were narrow-spectrum, and provider-ordered viral testing correlated with decreased prescribing of narrow-spectrum antibiotics, regardless of the test result. In the inpatient setting, 73% of all viral tests performed were a molecular respiratory panel, broad-spectrum antibiotics (empiric therapy) were the most frequently administered, and a negative provider-ordered viral test increased broad-spectrum antibiotic administration. Regardless of setting, a positive provider-ordered viral test did not impact antibiotic administration practices, and most children had a tier 3 discharge diagnosis suggesting antibiotic therapy was unnecessary.

In the ED, we found that provider-ordered viral testing decreased the use of narrow-spectrum antibiotics, which may reflect the high prevalence of influenza antigen testing and tier 3 diagnoses. Concordant with our findings, Bonner et al^23^ conducted a randomized prospective trial in an Alabama pediatric ED and reported a 34% decreased risk (RR: 0.66; 95% CI: 0.45, 0.96) of receiving an antibiotic among children with rapid influenza antigen testing.^12^ We also found that provider-ordered viral testing in the ED did not lead to a decreased use of broad-spectrum antibiotics, and positive viral tests were not associated with antibiotic administration practices. These findings mirror three of four studies included in a meta-analysis^12,24–26^, along with a single-center study in Colorado,^27^ and a multi-center study in Europe^28^ evaluating the effect of respiratory viral testing and antibiotic prescribing in EDs. Discordant results across study findings may be ascribed to the various test options available, or the sensitivity and specificity of testing platforms used across institutions. We also showed nearly three-quarters of antibiotics prescribed in the ED were narrow-spectrum and were most frequently administered to children with a tier 2 diagnosis, where more than one-half of diagnoses were attributed to acute otitis media. The use of narrow-spectrum antibiotics is in compliance with the American Academy of Pediatrics clinical management guidelines for acute otitis media, which recommends amoxicillin (narrow-spectrum) as the first-line agent when an antibiotic is indicated.^31^ Prior studies have revealed broad-spectrum antibiotics account for more than half of antibiotics prescribed in EDs for respiratory tract infections in children.^5,29,30^ However, these studies included non-pediatric EDs, which prescribed macrolides (broad-spectrum antibiotic) more often than pediatric EDs.^30^ Discrepancies across studies also may be attributed to varying practices and adherence to antimicrobial stewardship programs. Core elements of antimicrobial stewardship are set by the Centers for Disease Control and Prevention,^32^ but program components are developed and implemented by each individual institution/center. Further studies evaluating correlations between antibiotic stewardship in the ED, provider-ordered viral testing, and antibiotic prescribing practices are needed.

Among hospitalized children, the lack of a provider-ordered test was associated with a higher frequency of broad-spectrum antibiotics. This finding is not surprising and may be explained by the practice of administering empiric antibiotics (broad-spectrum) when there is suspicion of a bacterial infection, but the bacterial etiology is not yet known. Studies evaluating provider-ordered testing and antibiotic practices in hospitalized children have heterogeneous findings,^33–35^ and a meta-analysis concluded viral testing did not influence antibiotic practices in hospitals.^13^ Similar to the ED, testing platform options and antimicrobial stewardship practices and adherence might explain the variable impact of viral testing on antibiotic practices across studies. In our study, broad-spectrum antibiotics were the most common type of antibiotic used in the inpatient setting, with administration in both tier 1 and 3 diagnoses. Because we only assessed active and completed antibiotics, we were unable to determine whether empiric antibiotics were stopped among children with a tier 3 diagnosis once provider-ordered viral and bacterial test results were reported. Additional studies assessing the impact of provider-ordered testing on halting empiric antibiotic treatment of inpatients are needed to define and optimize the value of respiratory pathogen testing on antibiotic use in this setting.

Our study results should be considered in context of some limitations. First, findings are from one ED and hospital and may reflect antibiotic stewardship strategies implemented at our institution, a caveat to generalization of practices to other institutions or settings, such as outpatient or urgent care facilities. Second, analyses were restricted to active or completed antibiotic use and did not include association between viral testing and halting of empiric (broad-spectrum) antibiotic therapy. Therefore, total number of antibiotics ordered during a child’s ED visit/hospitalization may be underestimated. Third, we were unable to assess whether inpatient unit, clinician years of experience, or provider type (eg, attending physician, resident, physician assistant, nurse practitioner) impacted the frequency of viral testing and antibiotic administration practices. Prior studies have shown that both of these factors are associated with differences in antibiotic prescribing practices.^36^ Finally, the study period reflects clinical viral testing and antibiotic prescribing and administration practices prior to the coronavirus disease 2019 (COVID-19) pandemic. Studies have recently reported that during the COVID-19 pandemic, changes in healthcare-seeking behaviors (ie, decreases in visits) led to a dramatic reduction of antibiotic prescriptions (31.6 to 6.4 prescriptions per 1,000 visits) in children for respiratory tract infections^37^; however, from April to June 2021, studies have shown a rebound in both respiratory tract infections and antibiotic prescriptions.^37^ As respiratory viruses have resurged and viral testing is more broadly available, additional studies are needed to evaluate the relative impact of viral testing, including severe acute respiratory syndrome coronavirus 2, on antibiotic administration practices among children.

## Conclusion

In conclusion, the influence of provider-ordered viral testing on antibiotic administration practices differs by setting and may be heavily influenced by the components of and adherence to antimicrobial stewardship strategies within individual institutions. Future research should address the impact of provider-ordered testing on halting empiric antibiotics in inpatient settings and assess the combination of antimicrobial stewardship strategies and viral testing on antibiotic administration practices across settings.

## Supporting information

Rankin et al. supplementary material Rankin et al. supplementary material

## Data Availability

A deidentified dataset and coding will be made available upon request to the corresponding author.

## Supplementary material

For supplementary material accompanying this paper visit http://doi.org/10.1017/ash.2024.24. click here to view supplementary material
