# Supplementary material for: Provider-ordered viral testing and antibiotic administration practices among children with acute respiratory infections across healthcare settings in Nashville, Tennessee
Source: Antimicrob Steward Healthc Epidemiol. 2024 Mar 6;4(1):e29. doi: 10.1017/ash.2024.24 (PMC10945942; doi:10.1017/ash.2024.24)
Supplement: Rankin et al. supplementary material [file S2732494X2400024Xsup001.docx]

**Supplemental Online Content**

**eTable 1.** Classification of Antibiotics Administered During a Child’s Emergency Department Visit or Hospitalization, Nashville, Tennessee, November 2017-February 2020

**eFigure 1.** Assessment of Confounders using Directed Acyclic Graph (DAG) Theory

**eFigure 2.** Consort Diagram of Children (>30 days-17 years) Enrolled in an Emergency Department and Hospitalized with Acute Respiratory Illness, Nashville, Tennessee, November 2017-February 2020

**eTable 2.** Knot Placements for Continuous Variables with Restricted Cubic Splines, by Healthcare Setting

**eTable 3.** Provider-Ordered Viral Testing Results among Children (>30 days-17 years) with Acute Respiratory Illness, by Setting and Antibiotic Administration, Nashville, Tennessee, November 2017-February 2020

| eTable 1. Classification of Antibiotics Administered During a Child’s Emergency Department Visit or Hospitalization, Nashville, Tennessee, November 2017-February 2020 | |
| --- | --- |
| None Administered | No antibiotics were prescribed/administered during the child’s ED visit/hospital stay |
| Narrow-Spectrum | Penicillin   - Penicillin, Amoxicillin, Ampicillin, Nafcillin   1^st^ generation cephalosporins:   - Cefazolin, Cefadroxil, Cephalexin, Cephalothin, Cephapirin, Cephradine   Sulfonamides   - Bactrim, Bactrim DS, Cotrim, Septra, Sulfadiazine, Pediazole, Trimethoprim/Sulfametoxazole, Erythromycin/Sulfisoxazole, Septra DS, Sulfatrim   Tetracyclines   - Minocycline, Doxycycline, Sarecycline, Eravacycline, Oxytetracycline, Tetracycline   Gentamicin  Metronidazole |
| Broad-Spectrum | Amoxicillin-Clavulanate  Advanced generation cephalosporins:   - Cefoxitin, Cefotetan, Cefmetazole, Cefprozil, Cefaclor, Cefuroxime, Ceftriaxone, Cefepime, Cefotaxime   Ceftazidime, Cefixime, Cefdinir  Clindamycin  Quinolones   - Ciprofloxacin, Levofloxacin, Ofloxacin, Moxifloxacin, Gemifloxacin   Macrolides   - Erythromycin, Azithromycin, Clarithromycin, Fidaxomicin, Vancomycin, Tobramycin   Linezolid  Piperacillin/Tazobactam  Daptomycin |

eFigure 1. Assessment of Confounders using Directed Acyclic Graph (DAG) Theory. The minimally sufficient adjustment set for estimating the total effect of provider-ordered viral testing on antibiotic administration included: age, illness duration, illness severity, insurance, maximum temperature, pulmonary symptoms, time of year, and underlying medical conditions.

| eTable 2. Knot Placements for Continuous Variables with Restricted Cubic Splines, by Healthcare Setting | | | |
| --- | --- | --- | --- |
| Covariate | No. of Knots | Knot Values for ED | Knot Values for Inpatient |
| Age, years | 4 | 0.32  1.69  5.05  14.06 | 0.11  0.8  3.02  12.83 |
| Illness Duration, days | 3 | 0  2  6 | 1  3  7 |
| Respiratory Rate, bpm | 4 | 18  24  30  48 | 20  32  42  64 |
| Heart Rate, bpm | 4 | 88  125  148  182 | 102  141  162  191 |
| Oxygen Saturation, % | 3 | 96  99  100 | 90  97  100 |
|  | | | |
|  | | | |

eFigure 2. Consort Diagram of Children (>30 days-17 years) Enrolled in an Emergency Department and Hospitalized with Acute Respiratory Illness, Nashville, Tennessee, November 2017-February 2020

Abbreviations: ARI, acute respiratory illness

Footnote: ^a^total enrolled include children with multiple ARI visits ≤14 days apart; ^b^390 children had 2 ARI visits ≥90 days apart and 65 children ≥3 ARI visits ≥90 days apart; ^c^241 children enrolled had 2 ARI visits ≥90 days apart and 34 children had ≥3 ARI visits ≥90 days apart were in the emergency department; ^d^149 children had 2 ARI visits ≥90 days apart and 31 had 3 ARI visits ≥90 days were hospitalized.

| eTable 3. Provider-Ordered Viral Testing Results among Children (>30 days-17 years) with Acute Respiratory Illness, by Setting and Antibiotic Administration, Nashville, Tennessee, November 2017-February 2020 | | | | |
| --- | --- | --- | --- | --- |
|  | Emergency Department | | Inpatient | |
|  | Antibiotics (n=432)  N (%) | No Antibiotics  (n=2184)  N (%) | Antibiotics  (n=334)  N (%) | No Antibiotics  (n=1157)  N (%) |
| *Rapid Antigen Test, positive* |  |  |  |  |
| Rapid influenza | 112 (62) | 589 (62) | 54 (65) | 159 (62) |
| Rapid RSV | 1 (33) | 15 (60) | 13 (57) | 51 (54) |
| *Molecular Test, positive* |  |  |  |  |
| Adenovirus | 2 (8) | 18 (12) | 16 (9) | 42 (8) |
| Influenza | 2 (8) | 15 (10) | 14 (8) | 37 (7) |
| RSV | 3 (12) | 20 (13) | 28 (16) | 122 (24) |
| Rhinovirus/enterovirus | 6 (24) | 57 (37) | 49 (28) | 190 (37) |
| Parainfluenza  (Types 1-4) | 8 (32) | 21 (14) | 13 (7) | 32 (6) |
| Human metapneumovirus | 1 (4) | 7 (5) | 9 (5) | 36 (7) |
| Coronaviruses (endemic) | 1 (4) | 15 (10) | 10 (6) | 25 (5) |
| *Mycoplasma pneumoniae* | 1 (4) | 1 (1) | 18 (10) | 3 (1) |
| *Bordetella parapertussis* | 0 | 0 | 0 | 0 |
| *Legionella pneumophilia* | 0 | 0 | 0 | 0 |
| *Chlamydophilia pneumoniae* | 0 | 0 | 0 | 0 |
